# Asprosin in health and disease, a new glucose sensor with central and peripheral metabolic effects

**DOI:** 10.3389/fendo.2022.1101091

**Published:** 2023-01-05

**Authors:** Mariam Farrag, Djedjiga Ait Eldjoudi, María González-Rodríguez, Alfonso Cordero-Barreal, Clara Ruiz-Fernández, Maurizio Capuozzo, Miguel Angel González-Gay, Antonio Mera, Francisca Lago, Ahmed Soffar, Amina Essawy, Jesus Pino, Yousof Farrag, Oreste Gualillo

**Affiliations:** ^1^ SERGAS (Servizo Galego de Saude), NEIRID Lab (Neuroendocrine Interactions in Rheumatology and Inflammatory Diseases), Research Laboratory 9, IDIS (Instituto de Investigación Sanitaria de Santiago), Santiago University Clinical Hospital, Santiago de Compostela, Spain; ^2^ Euro-Mediterranean Master in neuroscience and Biotechnology, Faculty of Science, Alexandria University, Alexandria, Egypt; ^3^ International PhD School of the University of Santiago de Compostela (EDIUS), Doctoral Program in Drug Research and Development, Santiago de Compostela, Spain; ^4^ International PhD School of the University of Santiago de Compostela (EDIUS), Doctoral Program in Medicine Clinical Research, Santiago de Compostela, Spain; ^5^ National Health Service, Local Health Authority ASL 3 Napoli Sud, Department of Pharmacy, Naples, Italy; ^6^ Hospital Universitario Marqués de Valdecilla, Epidemiology, Genetics and Atherosclerosis Research Group on Systemic Inflammatory Diseases, IDIVAL, University of Cantabria, Santander, Cantabria, Spain; ^7^ SERGAS, Santiago University Clinical Hospital, Division of Rheumatology, Santiago de Compostela, Spain; ^8^ SERGAS (Servizo Galego de Saude), IDIS (Instituto de Investigación Sanitaria de Santiago), Molecular and Cellular Cardiology Lab, Research Laboratory 7, Santiago University Clinical Hospital, Santiago de Compostela, Spain; ^9^ Faculty of Science, Alexandria University, Alexandria, Egypt

**Keywords:** asprosin, adipokines, metabolic diseases, obesity, diabetes, PCOS

## Abstract

Adipose tissue malfunction leads to altered adipokine secretion which might consequently contribute to an array of metabolic diseases spectrum including obesity, diabetes mellitus, and cardiovascular disorders. Asprosin is a novel diabetogenic adipokine classified as a caudamin hormone protein. This adipokine is released from white adipose tissue during fasting and elicits glucogenic and orexigenic effects. Although white adipose tissue is the dominant source for this multitask adipokine, other tissues also may produce asprosin such as salivary glands, pancreatic B-cells, and cartilage. Significantly, plasma asprosin levels link to glucose metabolism, lipid profile, insulin resistance (IR), and β-cell function. Indeed, asprosin exhibits a potent role in the metabolic process, induces hepatic glucose production, and influences appetite behavior. Clinical and preclinical research showed dysregulated levels of circulating asprosin in several metabolic diseases including obesity, type 2 diabetes mellitus (T2DM), polycystic ovarian syndrome (PCOS), non-alcoholic fatty liver (NAFLD), and several types of cancer. This review provides a comprehensive overview of the asprosin role in the etiology and pathophysiological manifestations of these conditions. Asprosin could be a promising candidate for both novel pharmacological treatment strategies and diagnostic tools, although developing a better understanding of its function and signaling pathways is still needed.

## 1 Introduction

Adipose tissue operates as a central energy storage endocrine organ that produces a diversity of bioactive mediators known as adipokines (adipose-derived secreted factors) which possess proinflammatory or anti-inflammatory effects (1). Adipokines are mainly, but not exclusively, produced by lipid-rich cells termed adipocytes located in the white adipose tissue; the predominant type of fat in mammals (2). They can easily flow to the systemic circulation and exert their actions through an inter-cell communication network (autocrine, paracrine, endocrine). Moreover, they maintain the regulation of various aspects of the normal metabolic processes in the human body including glucose and lipid homeostasis, insulin sensitivity, and inflammatory response.

Metabolic syndrome (MetS) is a natural consequence of many risk factors including a sedentary lifestyle, uncontrolled nutrition, and excessive adiposity (3). Various scientific evidence suggests that obesity is the starting point of many metabolism-related diseases such as type 2 diabetes mellitus (T2DM), cardiovascular diseases, polycystic ovary syndrome (PCOS), and inflammatory rheumatic disorders (4, 5). Obesity usually provokes pathological acceleration of adipose tissue remodeling *via* alterations in the size (hypertrophy) and number (hyperplasia) of adipocytes (6, 7). Thus, it further causes adiposopathy, represented as an imbalance in the production and release of adipokines and consequent metabolic and cardiovascular disturbances (8, 9). During the past two decades, numerous profiling studies have identified growing number of adipokines and their roles in several signaling cascades in healthy and pathological conditions (10).

This review aimed to briefly discuss the discovery, synthesis, expression of asprosin and its cognate receptors. Additionally, it puts a spotlight on the multifunctional activity of asprosin and assesses the circulating levels of asprosin in several metabolism-related diseases obesity, T2DM, PCOS, and cancer.

## 2 Novel caudamin member: discovery and biosynthesis

Asprosin is a novel adipokine that recently joined the newly established protein hormone subclass caudamin (10). This subclass is synthesized differently from traditional protein hormones and peptides (11). Typically, members of this subclass are derived from a C-terminus cleavage event that yields a large protein while the alternative N-terminal segment renders a non-hormonal protein that does not associate with the hormone realm in terms of biosynthetic origin, properties, and function (11). As a caudamin member, asprosin is synthesized by cleaving the C-terminus of a parent protein called profibrillin-1 protein by the proteolytic enzyme furin (11, 12). This cleavage produces the 320 kDa glycoprotein fibrillin-1 in addition to the novel peptide hormone asprosin. Originally, profibrillin-1 is encoded by exons 65 and 66 of the fibrillin-1 gene (FBN1) located on chromosome 15q21.1. Mammalian asprosin is approximately a 30 kDa and 140 amino acids long protein (12).

Asprosin was first discovered at Baylor College of medicine by Romere et al. in 2016 while examining individuals with an inherited rare disorder named neonatal progeroid syndrome (NPS). In this disease, an elevated metabolic rate, low appetite, and insulin resistance (IR) lead to a generalized loss of subcutaneous fat and markedly low body weight, the leading cardinal features of NPS. The researchers found that the truncated heterozygous mutant form of the FBN1 gene caused a profound decreased plasma asprosin levels in NPS patients (12, 13). The same study also reported that adult IR obese mice have pathologically enhanced levels of asprosin during fasting. A single injection of recombinant asprosin in mice may cause an immediate increase in blood glucose and hyperinsulinemia (12). In addition, studies on humans with insulin resistance (IR) and high-fat diet mice models confirm the significant reduction of pathologically elevated asprosin levels after treatment with specific asprosin antibodies (13). Asprosin neutralization was reported to cause a concurrent decrease in food intake and body weight in mouse model of metabolic syndrome (14). These findings resulted in the characterization of asprosin as an orexigenic and glucogenic hormone.

## 3 Asprosin secretion and expression

Higher expression of FBN1 mRNA was detected in white adipose tissue suggesting it as the dominant producer of asprosin (12). A preclinical study showed lower plasma asprosin level in Bscl2_/_ deficient mice previously exposed to genetic resection of adipose tissue (≈ 65% decrease in adipose tissue) (15). Furthermore, asprosin may have a regulatory role in the browning process of primary adipocytes. Asprosin overexpression in subcutaneous white adipose tissue was revealed to downregulate browning-specific markers (UCP1) and accelerate lipid deposition *via* inhibition of the nuclear factor erythroid 2–related factor 2 (Nrf2) pathway in high-fat diet mice (16).

Other organs also appeared to produce this glucogenic peptide such as the heart, liver, pancreas, stomach, skeletal muscles, lungs, and brain (12, 17). Recent studies reported measurable amounts of asprosin in different body fluids such as saliva, breast milk, urine, serum, and plasma (18–20). Interestingly, asprosin was found to be expressed in the ovaries, placenta and human cartilage (19, 21, 22). A strong correlation was reported between serum asprosin levels and a specific marker for cartilage degradation termed cartilage oligomeric matrix protein (COMP) in patients subjected to total hip replacement surgery (19).

In humans and mice, asprosin is secreted into circulation in nanomolar concentration (12). Upon secretion, asprosin traffics *via* the circulation and is then recruited by the liver where it binds to the surface of hepatocytes and promotes blood glucose elevation which is vital for survival and brain function. The increased glucose production may serve as an inhibitor of asprosin secretion *via* a negative feedback loop (12).

## 4 Asprosin receptors

After secretion, circulating asprosin was found to exert peripheral and central modulating effects (15, 23). It acts as glucogenic and appetite stimulant hormone *via* the olfactory receptor 4M1 (OR4M1) and a cell surface receptor termed protein tyrosine phosphatase receptor δ (Ptprd), respectively (15, 23). OR4M1 (rhodopsin family member) is considered the primary asprosin receptor in humans, while OLFR734 is the mouse ortholog. Circulating asprosin binds to the OLFR734 receptor on the surface of hepatocytes to promote hepatic glucose production *via* activation of cyclic adenosine monophosphate (cAMP) second messenger system dependent protein kinase A (PKA) signaling pathway (12). OLFR734 is markedly distributed in the testis, liver, kidney, olfactory epithelium tissue, and olfactory bulb (15).

**Figure 1:**
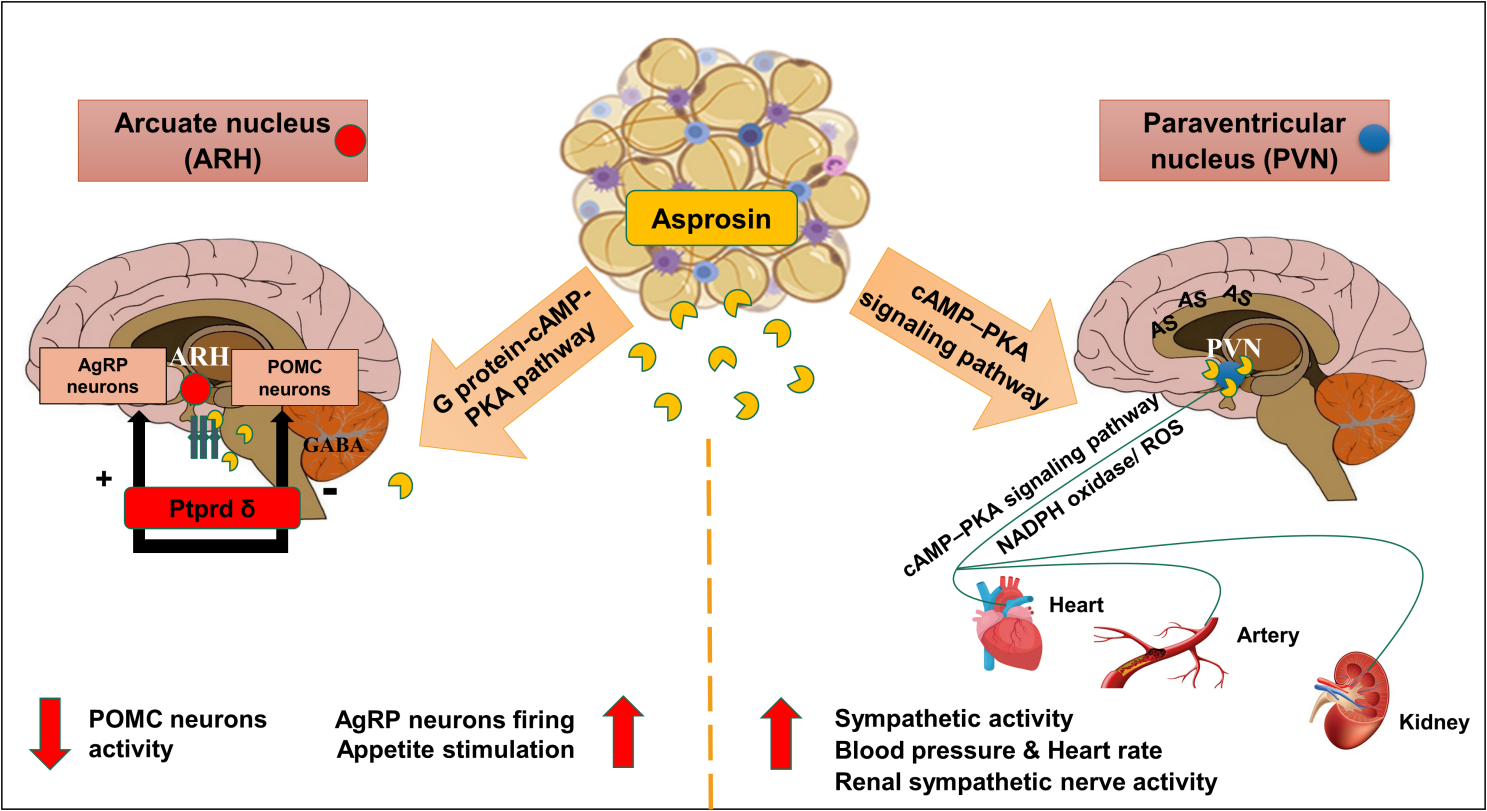
Schematic diagram of the central effects of asprosin in hypothalamic nuclei. Asprosin exerts its appetite stimulating effect by acting mainly *via* Protein tyrosine phosphatase receptor δ (Ptprd) in; Hypothalamic arcuate nucleus (ARH) causing stimulation of agouti-related protein neurons (AgRP) and inhibition of Pro-opiomelanocortin neurons (POMC). In paraventricular nucleus (PVN), asprosin increases the sympathetic outflow, blood pressure, and heart rate *via* promoting the cyclic adenosine monophosphate (cAMP) level, adenylyl cyclase (AC), and protein kinase A (PKA) activity mediated by nicotinamide adenine dinucleotide phosphate oxidase (NADPH oxidase) activity and superoxide production (ROS). (The illustration was created using the software: PowerPoint).

Centrally, asprosin hormone can pass across the blood-brain barrier and then activates the feeding neural circuitry to stimulate appetite (13). In the hypothalamic arcuate nucleus (ARH), asprosin acts directly on hunger-stimulating neurons known as agouti-related peptide neurons (AgRP) *via* the G protein-cAMP-PKA pathway (**Figure 1**) (13, 24). As previously mentioned, neutralization of serum asprosin with a monoclonal antibody in obese mice with pathologically elevated asprosin levels resulted in a decreased blood asprosin levels combined with reduced appetite and improved glycemic profile (13).

Simultaneously, asprosin was found to inhibit 85% of the appetite-suppressing neurons population pro-opiomelanocortin (POMC) in γ-Aminobutyric acid (GABA) dependent manner (13). Indeed, this inhibition can indirectly stimulate appetite, and adiposity accumulation (12, 13, 24). Recently, asprosin was found to have a high affinity to the cell surface receptor Ptprd, highly expressed in the hypothalamic AgRP neurons (23, 25). A preclinical study illustrated a significant reduction of appetite, blood glucose levels, and leanness after genetic excision of Ptprd receptors from AgRP neurons which suggested this receptor to be the orexigenic asprosin receptor (23). Notably, Ptprd membrane-bound receptors were not detected in the liver tissue which confirmed the absence of Ptprd role in the glucogenic function of asprosin (23).

As illustrated before, cAMP–PKA pathway is essential in AgRP neuronal activation that contributes to the orexigenic function of asprosin. This signaling pathway may also be implicated in the cardiovascular-related effects of asprosin. Recently, high expression levels of asprosin mRNA and protein were detected in another hypothalamic nucleus known as paraventricular nucleus (PVN) (26). Interestingly, asprosin induced the sympathetic outflow in the PVN *via* activation of the cAMP–PKA signaling pathway mediated by NADPH oxidase activation and subsequent reactive oxygen species (ROS) production (**Figure 1**) (26). Microinjection of asprosin protein in PVN of adult Sprague-Dawley *rats* caused increasing levels of heart rate, mean arterial pressure, and renal sympathetic nerve activity in a dose-dependent manner (26). However, neutralization of the endogenous asprosin with a specific antibody eliminated the previous asprosin-dependent actions (26).

Below the hypothalamus at the base of the brain, a master endocrine pituitary gland is located and charges several essential hormones. Abnormal excessive secretion of growth hormone from this gland due to pituitary cell adenoma develops a rare disease known as Acromegaly (AC). AC patients usually express uncontrolled glucose-lipid metabolism (insulin resistance, diabetes) with subsequent dysregulation of adipose tissue function and secretion. Patients with AC who have higher blood glucose and decreased fat mass were found to have significantly lower serum asprosin concentrations compared to healthy controls (27). Central administration of asprosin was reported to stimulate the hypothalamic-pituitary-testicular axis and significantly increase serum levels of pituitary-secreted hormones: luteinizing hormone (LH), follicle-stimulating hormone (FSH), and testosterone in rats (28).

## 5 Asprosin role in inflammatory response and cardiac related disease

**Figure 2:**
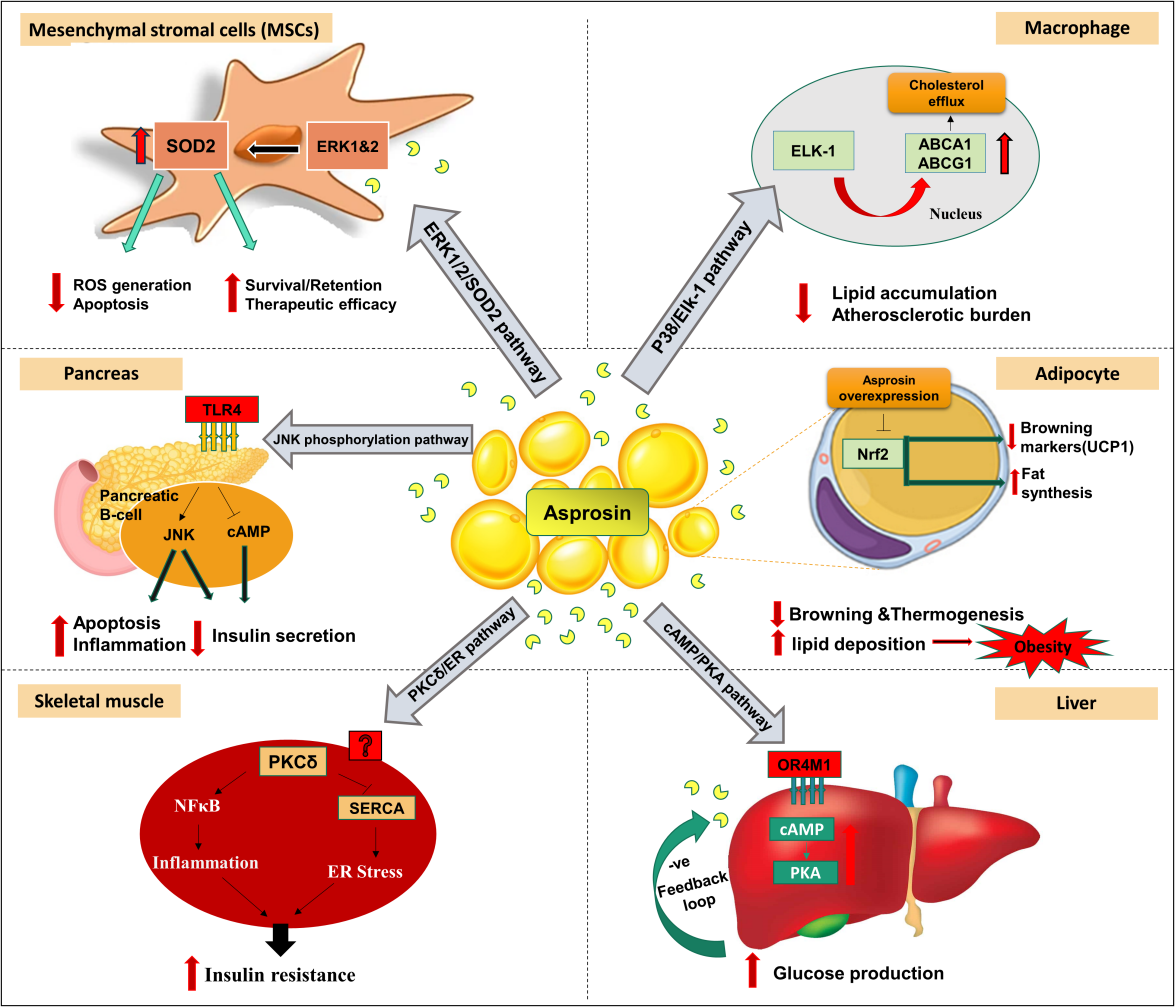
Signaling pathways and reported peripheral effects of asprosin in different organs, tissues, cells *in vivo* and *in vitro* models. In the liver, asprosin increased glucose production *via* a G-protein coupled receptor OR4M1 in the human (OLFR734 the mouse orthologue) through activation of cyclic adenosine monophosphate (cAMP)/protein kinase A (PKA) pathway. In pancreas, asprosin promotes inflammation, islet β-cells dysfunction and apoptosis *via* activation of toll-like receptor 4 (TLR4) expression and c-Jun N-terminal kinases (JNK) phosphorylation. In skeletal muscle caused insulin sensitivity impairment *via* activation of protein kinase C-delta (PKCδ)-associated endoplasmic reticulum (ER) stress/inflammation pathways. Overexpression of asprosin in adipocytes downregulated browning-specific markers (UCP1) and accelerated lipid deposition *via* inhibition of the nuclear factor erythroid 2–related factor 2 (Nrf2) pathway. Asprosin regulates the function and survival of mesenchymal stromal cells (MSC) against ROS generation and apoptosis *via* upregulation of superoxide dismutase 2 (SOD2) protein expression and the activation of extracellular signal-regulated kinase 1/2 (ERK1/2)-SOD2 pathway. Asprosin alleviated the macrophage’s inflammatory response and atherosclerotic burden *via* upregulating ATP-binding cassette transporters A1 (ABCA1) and ABCG1 by the stimulation of the P38/ETS-like transcription factor (Elk-1) pathway. Additional abbreviations within the figure: Sarco/endoplasmic reticulum Ca2+-ATPase (SERCA); Nuclear factor kappa-light-chain-enhancer of activated B cells (NFκB). (The illustration was created using the software: PowerPoint).

As a characteristic feature of MetS, obesity and diabetes are closely related to severe chronic inflammation (29). *In vitro* studies have illustrated that asprosin may exert a pro-inflammatory role in pancreatic β-cells and skeletal muscle cells (**Figure 2**) (30–32). Palmitate is a saturated fatty acid that promotes IR and inflammation in peripheral tissue (33). Palmitate treatment of mouse insulinoma MIN6 cells and primary human islet caused significant expression of asprosin along with reduced insulin secretion and cell viability (30). This treatment resulted in the release of inflammatory markers such as tumor necrosis factor alpha (TNF-α) and monocyte chemoattractant protein-1 (MCP-1), activation of nuclear factor kappa-light-chain-enhancer of activated B cells (NFκB) phosphorylation, apoptosis, and impaired insulin secretion (30). Moreover, asprosin promoted inflammation, islet β-cells dysfunction and apoptosis *via* activation of toll-like receptor 4 (TLR4) expression and JNK phosphorylation in a dose-dependent manner (30). Furthermore, asprosin showed activation of protein kinase C-δ (PKCδ)-associated endoplasmic reticulum (ER) stress/inflammation pathways in skeletal muscle causing insulin sensitivity impairment (31). Primarily, β‐cell dysfunction affects insulin production and leads to hyperglycemia (34). Asprosin stimulated β‐cell apoptosis *via* increasing the expression of caspase3 and inhibiting β‐cell autophagy *via* disrupting the AMP-activated protein kinase (AMPK) ‐ mechanistic target of rapamycin (mTOR) pathway (35).

A recent study illustrated the role of asprosin in alleviating the macrophage’s inflammatory response and inducing atheroprotection effect (36). Asprosin upregulated ATP-binding cassette transporters A1 (ABCA1) and ABCG1 *via* the stimulation of the P38/Elk-1 pathway with subsequent intracellular cholesterol efflux (**Figure 2**) (36). This reduced lipid accumulation and atherosclerotic lesion progression. In addition, asprosin downregulated the expression of inflammatory cytokines Interleukin 1 beta (IL-1β) and IL-6 (36).

An *in vivo* study suggested that asprosin can regulate the function and survival of mesenchymal stromal cells (MSC) in improving cardiac function (myocardial infarction) (37). In the ischemic microenvironment, asprosin protected MSC against ROS generation and apoptosis (37). This cytoprotective effect was executed *via* upregulation of SOD2 protein expression and activating the ERK1/2-SOD2 pathway (**Figure 2**) (37). Similarly, a clinical study reported lower adverse cardiovascular events in dilated cardiac myopathy patients with higher asprosin levels compared to lower asprosin patients (38). Furthermore, the same investigation also revealed that, under hypoxic conditions, asprosin directly induced cardio-protective effects on H9c2 cardio myoblasts accompanied by recovery of the mitochondrial respiration process (38).

## 6 Asprosin role in obesity and obesity-related disorders

Obesity is considered the driving force of various adverse metabolic consequences such as IR which causes impaired glucose tolerance over time and consequently T2DM. Its prevalence increases due to the obesogenic environment upon insufficient physical activity and the disruption in both dietary intake and energy expenditure balance.

Plasma asprosin was reported to be pathologically increased in obese children and adults (18, 39–41) (**Table 1**). The available data regarding the role of plasma asprosin levels in childhood obesity is still limited (39, 41). Two studies documented significantly lower asprosin serum levels in obese boys relative to obese girls proposing the involvement of sex differences in asprosin effects in pediatric obesity (43, 44). Conversely, Silistre and coworkers observed significantly higher serum levels of asprosin in Turkish obese children with no significant differences regarding gender variability which agrees with data from Wang et al. on obese adults (40, 41).

**Table 1:** Main findings of clinical trials investigating circulating asprosin levels and correlations in obesity.

Citation	Cohort	Target population (Study group)	n, gender	Main finding & Correlations
Wang et al. (41)	Obesity	Adult Obese receiving bariatric surgery (36-47 y)	174 females Obese patients (n = 117) Non-obese controls (n = 57)	⇑ Serum asprosin in obese than in non-obese subjects. ⇓ Serum asprosin after 6 months of bariatric surgery.
Cantay et al. (42)		Severe obesity patients (underwent sleeve gastrectomy)	74 subjects (19 m, 55 f) Obese + sleeve gastrectomy (n = 37) Obese + cholecystectomy (n = 37) Control group (n = 37)	⇑ asprosin levels and immunoreactivity in adipose tissue of obese patients. ⇓ Serum asprosin after 6 months of bariatric surgery.
Long et al. (43)	Obesity	Obese Chinese children (6-14 y).	87 children (49 boys, 38 girls) Obese (n = 40) Normal weight (n = 47)	⇓ Serum asprosin in obese children than in the normal-weight controls. ⇓ Serum asprosin in boys compared to girls, particularly in the obese group.
Wang et al. (39)	Cross-sectional study	Obese Chinese children	119 children (76 boys, 43 girls) Obese (n = 79) Lean controls (n = 40)	⇑ Serum asprosin in mild to moderately obese and severely obese groups than control groups, but asprosin with no differences between subgroups.
Silistre et al. (40)	Childhood obesity Cross sectional design	Obese Turkish children	158 children Obese (n = 44) Overweight (n = 54) Normal weight (n = 60)	⇑ Serum asprosin in obese group compared to the normal weight group with no gender differences.
Corica et al. (44)	Childhood obesity cross-sectional, case-controlled	Obese Caucasian children and adolescents (5-16 y)	67 subjects Obese Caucasian children (n = 43) (21 m - 22 f) Normal weight controls (n = 24)	⇓ Fasting serum asprosin in obese children compared to the lean controls ⇓ Fasting serum asprosin in boys than in girls. A negative correlation between serum asprosin and BMI without correlation with IR.
Corica et al. (45)	Cross-sectional study	Caucasian children and adolescents with obesity (5-16 y)	79 subjects (40 m, 39 f)	No significant difference between fasting and 2h-postprandial asprosin levels in obese non-diabetic group. ⇑ FBG levels in patients with significant increase in 2h-postprandial asprosin level compared to the other group which had decreased amounts. A positive correlation of asprosin with HOMA-IR, fasting glycaemia.
Ugur et al. (18)	Obesity	Adult obese patients	116 volunteers Low weight (n = 8) Normal weight (n = 44) Overweight (n = 19) Class I (n = 10) Class II (n = 13) Class III (n = 22)	⇑ Saliva and serum asprosin in class III obese group compared to other groups with the lowest concentrations in the underweight individuals.

A positive correlation between asprosin concentration and the homeostasis model of IR (HOMA-IR) has been observed (39, 41, 46, 47). Increased circulating asprosin levels were reported in IR obese children compared to the non-IR obese group (39), however, another study reported a negative correlation (43). Notably, salivary and blood asprosin concentrations were found elevated in class III obese subjects compared to the underweight group (18).

Prader–Willi syndrome (PWS) is a rare genetic disease that mainly affects the child’s metabolism and is characterized by uncontrolled feeding behavior with the development of severe obesity (48). A recent study found no differences in the asprosin fasting levels in children with PWS compared to overweight and standard weight groups (49). The same study also observed no significant differences between fasting and postprandial plasma asprosin levels in PWS children and the normal body mass index (BMI) controls (49). This finding agrees with a previous study which found increased fasting blood glucose (FBG) levels, but not the asprosin, in obese non-diabetic children who had a significant increase in 2h-postprandial asprosin level (45).

There is evidence suggesting an association between asprosin secreted levels and weight loss extent caused by bariatric surgery such as sleeve gastrectomy or cholecystectomy. Two studies reported significant decrease of serum asprosin levels after six months of weight loss surgical intervention (41, 42). Concentrations of fasting asprosin were significantly higher in obese individuals than in normal BMI subjects with no significant sex differences (41). Notably, obese group was found to express high asprosin levels and immunoreactivity in adipose tissue (42).

Regular exercise training remains the best and the safest non-pharmacological way for obesity management and avoidance. A preclinical study on experimental T1DM rats injected with 65 mg/kg intraperitoneal single dose of streptozotocin (STZ) found decreased levels of asprosin, PKA, and transforming growth factor-β (TGF-β), however, elevated AMPK and protein kinase B (AKT) levels in the liver under aerobic exercise training (50). Thus, aerobic exercise might modulate the hepatic asprosin-dependent downstream signaling pathways (cAMP/PKA) that lead to adjustment of the hepatic glucose release in T1DM (50). Additionally, the previously detected lowering impact of aerobic exercise on plasma asprosin and insulin levels was observed after morning and evening training in either standard weight or overweight and obese groups (51). Furthermore, a significant decrease in the blood asprosin and waist-hip ratio was observed in young women with abdominal obesity and metabolic diseases after two months of “Nordic” walking training program (52). Similar findings were noticed in obese sedentary men upon ending three months of different resistance training methods (traditional, circular, and interval) (53).

Asprosin hormone is closely related to non-alcoholic fatty liver disease (NAFLD), although the exact role remains unclear. NAFLD is the most common chronic liver complication among adult and pediatric obese subjects (**Table 2**). NAFLD Patients are usually susceptible to increased risk of IR and diabetes (59). The plasma asprosin levels were notably higher in obese Chinese children with NAFLD than the lean group with significant differences in liver enzymes alanine aminotransferase (ALT) and aspartate aminotransferase (AST) (55). Similarly, increased serum asprosin levels were detected in NAFLD adults (age > 18) with a positive correlation to HOMA-IR, FBG, triglycerides (TG), and serum albumin (ALB) (54).

**Table 2:** Main findings of clinical trials investigating circulating asprosin levels and correlations in obesity related diseases.

Citation	Cohort	Target population (Study group)	n, gender	Main finding & Correlations
Ke et al. (54)	NAFLD	NAFLD (age > 18 years)	93 subjects NAFLD patients (n = 43) Healthy controls (n = 50)	⇑ serum asprosin in patients with NAFLD compared to healthy control group. A positive correlation between plasma asprosin and HOMA-IR, FBG, TG, and ALB.
Liu et al. (55)		Children obese patients with NAFLD (6-18 y)	110 subjects (71 boys, 39 girls) Obese children (n = 39) Obese children with NAFLD (n = 36) Lean controls (n = 35)	⇑ serum asprosin in obese children, particularly in NAFLD obese group compared to the lean group. A positive correlation between serum asprosin and BMI, FBG, ALT and TNF-*α*
Hu et al. (56)	AN	Chinese female (average age 15.6 y)	93 participants AN patients (n = 46) Healthy subjects (n = 47)	⇑ Plasma asprosin in AN compared to healthy control subjects. ⇓ Plasma asprosin levels decrease with long term duration of illness.
Jowik et al. (57)	AN	Adolescent girls with AN (11–18 y)	Normal weight (n = 29) Adolescents with AN in extremely low body weight (n = 44) After partial normalization of body weight (n = 44)	⇑ Plasma asprosin in AN patient with increasing their body weight. No significant difference in plasma asprosin levels in acute phase adolescent group compared to the healthy control group.
Ding et al. (58)	OSA	OSA	249 participants OSA patients (*n* = 152) Control subjects (*n* = 97)	⇑ serum asprosin concentrations in OSA patients compared to healthy controls. A positive correlation with BMI, FPG, HOMA-IR, and TG, and negatively correlated with HDL-C.

Obesity is a shared risk factor between metabolic diseases and a common sleeping disorder known as obstructive sleep apnea (OSA) (60). The prevalence of OSA is estimated to be more than 50% among obese patients, particularly those who have abdominal and central fat deposition (61). One clinical study including 191 male population diagnosed with OSA found significantly increased serum asprosin concentrations compared with healthy control subjects (**Table 2**) (58).

On the other hand, plasma asprosin levels in patients with anorexia nervosa (AN) who suffer from self-induced extreme weight loss and malnutrition were increased compared to the corresponding healthy controls (**Table 2**) (56). These higher levels were decreased with long term deficient energy intake and depletion of body fat (56). Similarly, a recent study observed that plasma asprosin in AN acute phase were elevated with increasing body weight and positively correlated to the eating disorder symptoms (57). However, the same study reported no significant differences in the plasma asprosin between adolescents in the acute phase and the corresponding healthy controls (57).

## 7 Asprosin in type 2 diabetes mellitus

Elevated asprosin levels are associated with insulin resistance and T2DM (**Table 3**). Up to now, numerous clinical studies have ascertained increased plasma asprosin concentrations among pre-diabetic cases and T2DM patients (62–65). A hospital-based observational study comprised of 170 individuals showed a significant elevation in the serum asprosin in newly diagnosed T2DM adult patients compared to the normal glucose tolerance individuals (62). These findings were independently correlated to triglyceride levels, FBG levels, and adiposity-related parameters: BMI, waist circumference (WC), and waist-to-height ratio (WHR) in T2DM and persons without diabetes (62). Li et al. data were also in agreement with the previous study but with no association with plasma triglyceride levels (65).

**Table 3:** Main findings of clinical trials investigating circulating asprosin levels and correlations in T2DM.

Citation	Cohort	Target population (Study group)	n, gender	Main finding & Correlations
Zhang et al. (62)	T2DM Case-control design	Newly diagnosed T2DM (25-70 y)	170 subjects (101 m, 69 f) T2DM adults (n = 84) NGT (n = 86).	⇑ Serum asprosin in newly diagnosed T2DM adult patients than NGT group. No significant differences in serum asprosin levels between men and women.
Wang et al. (46)	T2DM cross-sectional study	Newly diagnosed T2DM (27-75 y)	143 participants T2DM group (n = 51) IGR (n = 40) NGT (n = 52)	⇑ Plasma asprosin in nT2DM and IGR compared to NGT group especially in IGR participants.
Zhang et al. (63)	T2DM	T2DM	120 participants T2DM patients (n = 60) NGT (n = 60)	⇑ Fasting asprosin and postprandial asprosin in T2DM patients than in NGT controls. ⇓ Serum asprosin with the onset of the oral glucose tolerance test (75 gm) in NGT group but not in T2DM group.
Naiemian set al. (47)	Observational study	New onset T2DM (52-54 y)	194 adults (100 m, 94 f) Newly diagnosed T2DM (n = 97) Healthy individuals (n = 97)	⇑ Serum asprosin in T2DM patients compared to healthy group. A positive correlation between asprosin concentrations and insulin resistance (HOMA-IR), TG, hemoglobin A1c (HbA1c), BMI, FBG, TC/HDL-C ratio (atherosclerotic risk factor of cardiovascular diseases) in T2DM patients.
Timurkaan et al. (64)	Observational study	Newly diagnosed T2DM	120 participants T2DM patients (n = 60) (30 m, 30 f) Healthy controls (n = 60) (29 m, 31 f)	⇑ Asprosin levels in T2DM patients compared to healthy controls. A positive correlation between asprosin levels and BMI, HOMA-IR, insulin, and TG levels in T2DM group.
Gozel et al. (20)	Observational study	New diagnosed T2DM treated with metformin	60 individuals (33 m, 27 f) Newly identified T2DM (n = 30) Healthy volunteers (n = 30)	⇑ Blood asprosin levels in newly diagnosed T2DM patients compared with the healthy volunteers. ⇓ Blood asprosin levels in newly diagnosed T2DM group after three months treatment with metformin. ⇑ Salivary asprosin levels in newly diagnosed T2DM group.

A cross-sectional study demonstrated significantly elevated plasma asprosin levels in newly diagnosed T2DM cases and impaired glucose regulation (IGR) compared to the healthy controls with no sex or age differences (46). Notably, plasma asprosin concentrations correlated positively with HOMA-IR, blood glucose, and WC (46). Another study also reported considerably higher serum asprosin levels in T2DM patients during fasting and postprandial events than in normal glucose tolerance (NGT) subjects (63). In addition, the postprandial asprosin concentrations seemed to be lower than the fasting levels in the NGT group (63). The same study also reported a negative correlation of serum asprosin levels with blood glucose after 75 g oral glucose administration in the NGT group but not in T2DM (63). These findings indicated an apparent response of asprosin to glucose *variations* in T2DM patients that may contribute to T2DM onset. Furthermore, Significant elevation of the asprosin concentration was found in old diagnosed T2DM Iraqi patients but not in the newly diagnosed cases (66). Besides, newly diagnosed T2DM patients treated for three months with metformin or 24 weeks with 10 mg/day sodium-glucose co-transporter-2 (SGLT2) inhibitor called dapagliflozin showed reduced levels of plasma asprosin (20).

## 8 Asprosin in diabetic complications

Literature data showed a potential link between high circulating asprosin and uncontrolled diabetes that usually displays microvascular diabetic complications such as nephropathy, retinopathy, and peripheral neuropathy (**Table 4**). As expected, circulating asprosin levels were significantly enhanced in T2DM patients with diabetic complications (67, 68, 70, 71). Diabetic nephropathy (DN) (also known as diabetic kidney disease), a common severe complication of both types of diabetes mellitus, is considered a major cause of end-stage renal failure that seriously affects diabetic patients’ quality of life (76). Various clinical studies reported a significant association between elevated serum asprosin levels and DN (67, 69, 70). Zhang et al. measured the circulating asprosin levels in three groups: NGT, T2DM group with early-stage DN, and T2DM without DN (67). Notably, plasma asprosin was increased in DN and non-DN patients compared to the NGT group with the highest amount in the diabetic patients suffering from DN (67). In addition, asprosin concentrations seem closely related to the clinical parameters of the DN early stages: estimated glomerular filtration rate (e-GFR) and the urine albumin-creatinine ratio (UACR) (67). A positive correlation was found between asprosin plasma levels and UACR suggesting asprosin as an early clinical indicator for DN progression (68). These data seem to be consistent with a recent study in which a significant higher asprosin levels were reported in T2DM patients with macroalbuminuria than both microalbuminuria group and patients who had no signs of albuminuria among both men and women (77).

**Table 4:** Main findings of clinical trials investigating circulating asprosin levels and correlations in diabetic complications.

Citation	Cohort	Target population (Study group)	n, gender	Main finding & Correlations
Zhang et al. (67)	Cross-sectional study	DN (30 years & older)	105 Chinese subjects NGT (n = 30) T2DM + DN (n = 42) T2DM + early stage of DN (n = 33)	⇑ Significantly higher circulating asprosin in non-DN and DN groups than NGT group. DN group showed the highest levels. Circulating asprosin levels were closely associated with e-GFR and UACR and significantly increased ORs for early stage of DN.
Deng et al. (68)	Cross-sectional study,	T2DM with Microalbuminuria (Expected DN) (Mean age 61.6 ± 10.7 years)	207 patients with T2DM (120 m, 87 f) T2DM normoalbuminuria (n = 107) T2DM with microalbuminuria (n = 80) T2DM with macroalbuminuria (n = 20)	⇑ serum asprosin levels in T2DM patients with macroalbuminuria and microalbuminuria group compared with normoalbuminuria group. ⇑ Positive correlation between serum asprosin concentrations and creatinine, blood urea nitrogen (BUN) and UACR.
Wang et al. (69)	Cross-sectional study	T2DM with different stages of DN	284 subjects (152 m-132f) 212 T2DM adult patients (n = 212) Normal to mildly increased group (DN0) Moderately increased group (DN1) Severely increased group (DN2) Healthy controls (n = 72)	⇑ serum asprosin in all T2DM patients compared to controls. ⇑ serum asprosin in DN2 group compared to DN1 & DN0 groups.
Goodarzi et al. (70)	cCse–control study	T2DM patients & T2DM + DN	166 participants T2DM (n = 56) T2DM + DN (n = 54) Healthy volunteers (n = 56)	⇑ serum asprosin level in T2DM and T2DM + DN groups than healthy group. A positive correlation between asprosin and BMI and kidney function markers e GFR, UAE, and Creatinine in T2DM + DN patients.
Xu et al. (71)	Cross-sectional study	T2DM patients with different grades of DN	498 T2DM patients (298 m, 200 f) Microalbuminuria (n = 221) Massive albuminuria (n = 105) No signs of nephropathy (n = 172)	⇑ Serum asprosin in the massive albuminuria group compared to microalbuminuria group and patients with no signs of albuminuria. A positive correlation between serum asprosin and BMI, TG, diastolic blood pressure (DBP), AST, ALT, creatinine.
Oruc et al. (72)	Observational study	DRP	90 participants T2DM and DRP with single eye and cataract (n = 30) T2DM and cataract without DRP (n = 30) Healthy control (n = 30)	⇑ Blood and aqueous humor asprosin levels in patients with DRP compared with non DRP.
You et al. (73)	Cross-sectional single-center study	T2DM patients with PAD	114 subjects T2DM patients (n = 33) T2DM patients with PAD (n = 51) Healthy control (n = 30)	⇑ circulating asprosin in T2DM + PAD group compared to healthy control.
Deng et al. (74)	Observational study	T2DM patients with carotid plaque	180 subjects	⇑ serum asprosin in the T2DM with carotid plaque group compared to T2DM without carotid plaque group A positive correlation between serum asprosin and BMI, HOMA-IR.
Hong et al. (75)	Observational study	MetS (18-70 y)	293 participants (143 m,150 f) MetS patients (n = 131) Healthy subjects (n = 162)	⇑ serum asprosin in MetS patients compared to the healthy controls ⇑ Positive correlation between serum asprosin levels and metabolic components including BMI, WC, FPG, 2h-PG, HOMA-IR, TG.
Li et al. (65)	T2DM or PCOS	T2DM or PCOS	160 female subjects T2DM (n = 53) PCOS (n = 41) Healthy controls (n = 66)	⇑ Plasma asprosin in T2DM females than in healthy subjects ⇑ Plasma asprosin in PCOS subjects than in healthy subjects but lower than in T2DM subjects. Plasma asprosin showed positively correlated with FBG, HbA1c, HOMA-IR, LDL-c, Apolipoprotein B (APOB), APOE, and testosterone.

T2DM patients *are usually subjected to high-risk* of atherosclerotic plaques (74). Notably, a significant asprosin levels were detected in T2DM patients with carotid plaque compared to non-carotid plaque T2DM group (74). Furthermore, elevated plasma asprosin levels was reported in T2DM patients suffering from diabetic lower extremity peripheral artery disease (PAD) (73). Asprosin induced TGF-β signaling pathway activation that directly affects endothelial-to-mesenchymal transition (EndMT) (73).

Diabetic retinopathy (DRP), one major common cause of vision loss in diabetic people, was also found to be associated with circulating asprosin levels. DRP patients have been found to exhibit higher serum fasting asprosin values compared to non-diabetic retinopathy individuals or non-complicated cases of T2DM patients (72). Oruc et al. reported a significant increase in levels of serum asprosin and particular markers for oxidative stress: 4-hydroxynonenal (4-HNE) and 8-hydroxy-2”-deoxyguanosine (8-OHdG) in DRP patients (72). Interestingly, researchers have found a link between the asprosin production and central serous chorioretinopathy (CSCR), another retinopathic, yet non-diabetic, condition. It is an idiopathic retinal neurosensory disorder characterized by macular detachment with subsequent atrophy and unilateral vision loss (78). CSCR individuals exhibit a corrupted antioxidant defense system with a significant reduction of plasma antioxidative parameters (TAC and DHEA-S) (79). Celik and his team found significantly increased asprosin serum concentration in individuals with CSCR compared to the healthy subjects suggesting that this molecule might be implicated in the pathogenesis of CSCR (80).

*In vivo* study revealed decreased asprosin levels in a mouse model of neuropathic pain compared to healthy controls. In addition, treating these mice with recombinant asprosin yielded an anti-hypersensitivity effect suggesting an analgesic function of asprosin (81).

## 9 Asprosin in obstetrics and gynecology

During pregnancy, different adipokines are synthesized and secreted from the cord blood and placenta such as leptin, resistin and adiponectin having great effects on fetus development and metabolism (82, 83). In addition, maternal-related pathologies such as preeclampsia (PE) and gestational diabetes mellitus (GDM) are closely related to pre-gestational and gestational obesity (84, 85). Few published studies have addressed the role of asprosin during pregnancy and other gynecologic disorders (**Table 5**) (22). Asprosin concentration showed a possible impact on the incidence of diabetes developed during gestation.

**Table 5:** Main findings of clinical trials investigating circulating asprosin levels and correlations in obstetrics and gynecology.

Citation	Cohort	Target population (Study group)	n, gender	Main finding & Correlations
Alan et al. (86)	PCOS cross-sectional study	PCOS	156 females PCOS (n = 78) Healthy controls (n = 78)	⇑ Serum asprosin in PCOS than in healthy controls. Asprosin levels in positive correlation with HOMA-IR, BMI, free androgen index (FAI), and high sensitivity C-reactive protein (hs-CRP).
Chang et al. (87)		PCOS (18-34 y)	600 females PCOS (n = 444) Healthy controls (n = 156)	No difference of serum asprosin levels between PCOS females and the healthy controls.
Deniz et al. (88)	Case-control study	PCOS	60 participants PCOS (n = 30) Healthy controls (n = 30)	⇑ Blood asprosin in PCOS patients compared to normal control subjects.
Jiang et al. (89)		PCOS	170 women PCOS (n = 93) Healthy controls (n = 77)	⇓ asprosin level slightly in PCOS women with hyperandrogenism or IR compared to the non-hyperandrogenism, and non-IR control groups compared to healthy controls. A negative correlation between serum asprosin and BMI, HOMA-IR, and TG.
Baykus et al. (90)		Females with pathological pregnancies (18-35 y)	179 pregnant women GDM (n = 30) PE (n = 30) Severe PE (n = 30) IUGR (n = 30) MF (n = 29) healthy pregnant (n = 30)	⇑ Newborn cord blood & maternal blood asprosin in GDM, PE, SPE, and MF pregnant women compared to healthy pregnant women.
Zhong et al. (91)	Nested case-control study	Early diagnosed GDM (18-40 y)	80 pregnant women GDM pregnant women (n = 40) NGT controls (n = 40)	⇑ Plasma asprosin in pregnant females suffering from GDM and their offspring ⇑ Plasma asprosin in umbilical cord of neonates of GDM group than in NGT group.
Behrasi et al. (92)		Maternal obesity & Excessive gestational weight gain (EGWG)	60 women Pregnant women with normal weight (n = 30) Pregnant women with EGWG (n = 30)	⇑ umbilical cord blood asprosin levels were significantly higher in EGWG group compared to normal weight control group. No difference in maternal asprosin levels between both groups.
Hoffmann et al. (22)		Pregnant women with obesity/GDM	247 pregnant females	⇑ Maternal and fetal plasma asprosin concentrations in non-smoking obese and normal-weight mothers with GDM. A positive correlation between maternal insulin levels and placental asprosin in GDM patients. Direct correlation between maternal plasma asprosin amount and the number of exercises per week during pregnancy.
Leonard (93)	Observational study	Pre-menopausal women (Trained 201–825 min/week)	Healthy women (n = 32) Trained OC (n = 8) Trained Non-OC (n = 6) Untrained OC (n = 10) Untrained Non-OC (n = 8)	⇓ plasma asprosin in females using OC compared to non-OC. ⇑ plasma asprosin increased in the early follicular menstrual phase group compared to the mid-luteal and late follicular group, especially in OC users whereas the non-OC in the mid-luteal phase expressed the highest level.

Baykus and coworkers investigated the asprosin levels in newborn umbilical cord blood and maternal blood in 149 female patients with different forms of pathological pregnancies (90). They found a statistically significant increase in newborn cord blood and maternal blood asprosin levels in abnormal pregnancies encompassing GDM, PE, severe PE, and macrosomic fetuses (MF) compared with healthy pregnant women. However, the intrauterine growth retardation cases showed reduced asprosin levels (90). These observations might suggest that increased asprosin concentrations in pathological pregnancies could be a predisposing factor for PE and severe PE in pregnant women as it contributes to glucose homeostasis imbalance. Following the previous findings, another study also found elevated asprosin plasma levels in pregnant females suffering from GDM and their offspring (91).

Hoffmann et al. found in a placenta cohort study on 247 pregnant females a strong correlation between different metabolic parameters (obesity and GDM) and asprosin plasma levels and distribution in placental cells and tissues (22). Notably, this study reported significant asprosin concentrations in maternal and fetus plasma in non-smoking obese and normal-weight mothers with GDM (22). Interestingly, there was a direct correlation between maternal plasma asprosin amount and the number of exercises per week during pregnancy (22). Asprosin levels were also found significantly higher in the umbilical cord blood of pregnant women with maternal obesity than in the normal weight control group with no difference in maternal asprosin concentrations (92).

Moreover, some studies have speculated that obesity, in particular the central obesity, may be the primary metabolic insult in a common reproductive endocrinopathy mainly affecting women in childbearing age known as polycystic ovarian syndrome (PCOS) (94). Up to 70% of PCOS women markedly express obesity and IR which is considered as vital risks for PCOS pathogenesis (95). Indeed, PCOS is a key risk factor for other metabolic comorbidities such as cardiovascular diseases, T2DM, GDM, endometrial cancer as well as pregnancy-related complications (96).

The potential impact of asprosin in the etiology of PCOS is still controversial. Some studies addressed elevated levels of circulating asprosin in women with PCOS compared to the control subjects (65, 86, 88). In contrast, a cohort study of 444 women suffering from PCOS (18 to 34 years old) reported non-significant difference in the circulating asprosin between PCOS patients and the corresponding controls (87). A recent study reported partly congruent findings in which slightly lower asprosin levels were found in PCOS women with hyperandrogenism or IR compared to the non-hyperandrogenism and non-IR control groups (89).

Fasting plasma asprosin levels in pre-menopausal women was investigated during different stages of the menstrual cycle (early follicular, late follicular, and mid-luteal) (93). The findings demonstrated a reduction of fasting plasma asprosin in females using oral contraceptives (OC) compared to non-users (93). In addition, plasma asprosin increased in the early follicular menstrual phase compared to the mid-luteal and late follicular, especially in OC users whereas the non-OC users in the mid-luteal phase expressed the highest level (93). Hence, the menstrual cycle phase and the use of OC appeared important to be considered throughout investigating the concentration of circulating asprosin in women (93).

## 10 Asprosin in cancer

Currently, the view of cancer as a genetic condition has gradually been replaced by considering it a metabolic case. Emerging evidence supports a general hypothesis that cancer is a disease of impaired cellular energy metabolism regardless of its cellular or tissue origin (97). Cancer cells exhibit distinctive characteristics through different reprogrammed metabolic activities involving rapid glucose uptake and lipid metabolism dysregulation (98). Typically, Warburg effect represents the central adaptation mechanism to support the growth and the survival of tumor cells. In this circumstance, even under aerobic glycolysis, the enhanced rate of glucose uptake accompanied with preferential lactate production allows uncontrolled proliferation and maintenance of the tumor microenvironment (99).

Research studies suggested that many adipokines may promotes tumorigenesis and cancer progression through the enhancement of cell migration and proliferation and other anti-apoptosis pathways (100). However, only a few data are available regarding levels of the asprosin peptide in cancer. Interestingly, Kerslake et al. identified high expression levels of asprosin and its conjugate olfactory receptor OR4M1 in normal human ovaries and malignant ovarian tissues revealing its implication in the tumor microenvironment (21). Recently, another *in vitro* study of the same group reported differential regulation of genes following 100 nM asprosin treatment of the ovarian cancer cell line SKOV-3 (101). Additionally, asprosin induced ERK1/2 phosphorylation and altered numerous signaling pathways associated with cell communication and proliferation (101). The functionally expressed genes such as Cadherin 11 (CDH11) and Fc fragment of immunoglobulin G receptor IIa (FCGR2A) were previously reported to be implicated in the regulating of metastatic progression and chemotherapeutic response in different cancers and may also have an influence on Warburg effect and tumor microenvironment in ovarian cancer (101–103).

Kocaman et al. observed enhanced expression and immunoreactivity of asprosin in a paraffin embedded specimens of rare surface tumor known as malignant mesothelioma (MM) compared to reactive mesothelial hyperplasia (RMH) specimens as a control group (104). The same author also suggested in a recent study the possibility of using asprosin in the differentiation between two types of skin cancer which originate from hair follicles called basal cell carcinoma (BBC) and hair follicle trichoblastoma (105). Asprosin immunoreactivity is also significantly higher in BCC samples with no expression illustrated in trichoblastoma samples (105).

As mentioned before, asprosin causes hyperinsulinemia and IR which in turn could affect oxidant-antioxidant balance and increase the expression of Insulin-like growth factor 1 (IGF-1) (12, 46) which promotes cancer development and metastasis (106).

As cancer progresses, the majority of cancer patients might develop cancer-associated malnutrition termed cachexia syndrome (CACS) with prevalence rates varying from 9% to 85% based on the type and stage of the tumor causing short-term survival and impaired quality of life (107). It is characterized by systemic inflammation, energy imbalance, and involuntary loss of lean body mass, with or without adipose tissue wasting (108). During anorexia, adipose tissue depletes in response to nutritional deficit, but a degeneration of adipose tissue and skeletal muscle occurs in CACS (109). Significantly lower asprosin levels were observed in cancer patients suffering from anorexia, but not CACS, than in non-anorexic subjects (110). More research is needed to clarify the possible role of asprosin in cancer pathogenesis and progression.

## 11 Conclusion

Asprosin is a novel glucose sensor secreted mainly by white adipose tissue during fasting and has multifaceted metabolic roles either peripherally or centrally. The 3’ truncated mutation in the FBN1 gene leads to deficient asprosin expression and reduces the maintenance of healthy fat tissue. FBN1 gene, in line with its expressed protein asprosin, could exert different effects on health and diseased states. In the hypothalamus, asprosin activates two nuclei ARH and PVN triggering appetite stimulation and sympathetic flow, respectively. Peripherally, asprosin directly affects insulin signaling causing increased insulin resistance, inflammation, and endoplasmic reticulum stress. Since its discovery, asprosin has provided new insights into clinical diagnostic practice, particularly in higher-prevalence chronic diseases such as obesity and T2DM. Pathologically elevated asprosin levels might be a potential biomarker for early alteration in glucose metabolism in obese and diabetic cases. However, there are inconsistencies between the asprosin values of both obese children and obese adults. Besides, higher levels of asprosin could be a risk factor for developing diabetic complications such as diabetic nephropathy and retinopathy. As mentioned before, asprosin was positively correlated with insulin resistance, obesity, cholesterol metabolism, renal function, and inflammatory markers. Taken together, these data suggest a potential role for this adipokine in the metabolic process. All these findings open the doors for asprosin to be a new potential therapeutic target in an array of metabolic-related pathologies. However, more comprehensive studies are needed to explore the exact molecular mechanisms underlying the diverse effects of asprosin in physiological and pathological events.

## Author contributions

“Conceptualization, OG, DE, and MF; methodology, OG and AM, and MC; validation, FL, JP, and MG-G.; writing—original draft preparation, MF and YF; writing—review and editing, OG, MF, and YF; visualization, MF, MG-R, and AC-B; supervision, OG, YF, AS, and AE; funding acquisition, OG, JP, and FL. All authors contributed to the article and approved the submitted version.

## Glossary

**Table:**

4-HNE	4-hydroxynonenal
8-ohdg	8-hydroxy-2”- deoxyguanosine
ABCA1	ATP-binding cassette transporters A1
Agrp	Agouti-related peptide neurons
AKT	Protein kinase B
ALT	Alanine aminotransferase
AMPK	AMP-activated protein kinase
AN	Anorexia nervosa
APOB	Apolipoprotein B
ARH	Hypothalamic arcuate nucleus
AST	Aspartate aminotransferase
BMI	Body mass index
CACS	Cachexia syndrome
cAMP	Cyclic adenosine monophosphate
COMP	Cartilage oligomeric matrix protein
CSCR	Central serous chorioretinopathy
DCM	Dilated cardiomyopathy
DN	Diabetic nephropathy
DRP	Diabetic retinopathy
E-GFR	Estimated glomerular filtration rate
EGWG	Excessive gestational weight gain
EGWG	Excessive gestational weight gain
EndMT	endothelial–to–mesenchymal transition
ER	Endoplasmic reticulum
FAI	Free androgen index
FBG	Fasting blood glucose
FBN1	Fibrillin-1 gene
FSH	Follicle-Stimulating Hormone (FSH)
GABA	Γ-Aminobutyric acid
GDM	Gestational Diabetes Mellitus
Hba1c	Hemoglobin A1c
HOMA-IR	Homeostasis model of insulin resistance
HOMA-β	Homeostasis model assessment for β-cells function
IGR	Impaired glucose regulation
IL	Interleukin
IR	Insulin resistance
IUGR	Intrauterine growth retardation
LH	Luteinizing hormone
MCP-1	Monocyte chemoattractant protein-1
MF	Macrosomic fetuses
NAFLD	Non-alcoholic fatty liver disease
NGT	Normal glucose tolerance
NPS	Neonatal progeroid syndrome
Nrf2	nuclear factor erythroid 2–related factor 2
OSA	Obstructive sleep apnea
PAD	Peripheral artery disease
PCOS	Polycystic ovarian syndrome
PE	Preeclampsia
PKA	Protein kinase A
Pkcδ	Protein kinase C-δ
POMC	Pro-opiomelanocortin
Ptprd	Protein tyrosine phosphatase receptor δ
PVN	Paraventricular nucleus
PWS	Prader–Willi syndrome
ROS	Reactive oxygen species
SGLT2	Sodium-glucose co-transporter-2
T2DM	Type 2 diabetes mellitus
TG	Triglycerides
TGF-β	Transforming growth factor-β
TLR4	Toll-like receptor 4
TNF-α	Tumor necrosis factor alpha
UACR	Urine albumin-creatinine ratio;
WC	Waist circumference;
WHR	Waist-to-height ratio
